# Assessment of indoor radon levels at multiple floors of an apartment building in the historic center of Rome (Italy): a comprehensive study

**DOI:** 10.1007/s11356-024-35266-7

**Published:** 2024-10-21

**Authors:** Gaia Soldati, Maria Grazia Ciaccio, Valentina Cannelli, Antonio Piersanti, Gianfranco Galli

**Affiliations:** Istituto Nazionale Di Geofisica E Vulcanologia, Via Di Vigna Murata 605, 00143 Rome, Italy

**Keywords:** Indoor radon, Active monitoring, Residential radon, Radon risk assessment, Urban environment, Urban heat island

## Abstract

The urbanized area of Rome is largely built over volcanic deposits, characterized by a significant radionuclides content and consequently a high radon emanation potential. An accurate monitoring of workplaces and residential dwellings constitutes a first step towards mitigating the indoor radon exposure. Since radon diffusion dynamics involves complex interactions among many environmental parameters on different time scales, a proper assessment of radon concentration variations can be better achieved by means of active monitoring approaches. We present here the results of 1 year of continuous measurements conducted in six premises (five apartments and a basement) at different floors of the same building in the Esquilino district, in the historical center of Rome. Collecting annual series of radon concentration enables us to identify fluctuations over a seasonal scale, with radon generally decreasing in the warm season. The simultaneous tracking of different floors should cancel the influence of geogenic radon and of building characteristics like age, typology, and construction materials. While the basement shows the highest radon concentration, indicating a major contribution from the ground, we observe indoor radon levels comparable at all the upper floors, questioning the common belief that high-risk exposure be limited to the lowest storeys. The use of active devices makes it possible to discriminate between average indoor radon measured during the day and overnight, when residents are more likely to be at home. Our analysis provides the characterization of the dynamics of the gas emanation and transport inside the buildings and of its temporal fluctuations, in relation to the environmental and meteorological conditions. Since the experiment was performed in the Roman urban contest, we cannot ignore the specificity of the retrieved data, affected not only by endogenous factors like life habits relative to ventilation and conditioning of the apartments, but also by exogenous factors, among which the warmer microclimate compared to the surrounding suburban and rural areas, due to the effects of urbanization (urban heat island effect).

**Supplementary Information:**

The online version contains supplementary material available at 10.1007/s11356-024-35266-7.

## Introduction

Well before the discovery of radon in 1898, an unusually high rate of mortality from lung disease among young miners in the Schneeberg region in Saxony had been observed since the early sixteenth century. Only in 2009, the World Health Organization classified radon as a human carcinogen, estimated to be responsible for up to 15% of all lung cancers worldwide (WHO 2009). All outdoor and indoor air has some radon in it, but while in the atmosphere the gas quickly dissipates, it may build up in closed environments (homes, schools, and workplaces), where the greatest exposure for persons occurs; that makes radon an indoor air pollutant, while of a different nature compared to the conventional pollutants, exclusively anthropogenic.

We are generally more concerned about outdoor air pollution, which in modern cities has become a major issue and which is thought to be enhanced by global warming, since extreme heat and stagnant air increase the amount of ozone and particulate pollution. But temperature changes—as well as the variations of other meteorological parameters—may also impact indoor air pollution. In fact, weather conditions strongly influence both the exhalation of radon from the ground and its transport and convective circulation inside the buildings. It is also important to consider the further contribution to temperature due to the so-called urban heat island (UHI) effect (Mills et al. 2022; Oke 1973; Ren et al. 2023; Zhou et al. 2014). In fact, cities tend to be significantly hotter than suburban areas, due to passive (abundance of concrete, asphalt and closely spaced buildings creating a heat-trapping effect) and active (high density of heat emitters like all electric devices, heaters and conditioners, and combustion engines) effects. As a direct consequence, the health of city dwellers may suffer the synergistic impact of elevated temperatures and indoor radon pollution.

The primary source of radon is uranium content in rocks, so geology determines the production and distribution of radon in soils. We performed our experiment in the historic district of Esquilino, one of the 22 quarters of Rome, and the largest of its seven hills. The geology of this area mainly consists of the presence of volcanic sediments from the Colli Albani district (Marra and Rosa 1995). Actually, the structural-surface remains of the Alban-Hills and Sabatini-Mts. volcanic plateau are covered by many meters of anthropogenic deposits. The present landscape results from several human-driven geomorphic processes undertaken over almost three millennia (Luberti and Del Monte 2020). Various facies of tuff deposits have been used in construction, to produce building blocks, tombs, and amphitheaters, directly carved into the lithified pyroclastic deposits, and the deposits themselves have offered ideal foundation materials for the city’s growth (Funiciello et al. 2006; Marra et al. 2011). The Esquilino was born in the late nineteenth-century context: after Rome became the Italian capital (1871), there was an enormous building expansion to meet the housing needs due to the new role of the city and the imbalance between the availability of housing and population growth. The style of the Esquilino palaces sees the new buildings, 5–6 floors high, with vertical walls generally made of tuff masonry. The thermo-mechanical properties of tuffs made them a rather attractive building material; however, they are known for their radioactivity usually higher than other rocks, leading to higher exposure to the population (Righi and Bruzzi 2006; UNSCEAR 2000). This is a major problem for a city like Rome given that, according to the distribution of buildings on the different lithologies present on its territory, 57.2% of the urbanized sector within the GRA (Grande Raccordo Anulare, a ring-shaped motorway that encircles Rome) is built on volcanic deposits (Funiciello et al. 2008).

Although endogenous factors have the main role in the amount of radon released by soils, other factors, such as climatic ones, are essential in the diffusion process by which radon moves. The radon diffuses through rocks depending on the size of the pores and the pressure gradients across them or for natural geological features such as faults or fractures (Benà et al. 2023). Meteorological conditions are thought to be a key factor on radon migration, since rainfall, winds, and surface temperature dynamics induce pressure gradients that may influence radon transportation in porous media (Inan et al. 2012; Klusman and Webster 1981). The relative importance among the main relevant variables cannot be unequivocally discriminated; however, on the long timescales, radon anomalies are dominated by seasonal variations of surface temperature, generally positively correlated with them (the strong site-dependent nature of radon emissions prevents to generalize these findings).

Rome is the most populous municipality in Italy (2,748,109 inhabitants, dati.istat.it) and the third largest in the European Union after Berlin and Madrid. These data, combined with the fact that 24% of the territory of Rome is consumed soil, that a large part of it is waterproofed (91%), and the great irregularity of the dimensions of the streets and buildings as a result of the often disorderly growth of the city, makes the municipality of Rome particularly vulnerable to the consequences deriving from the impacts of climate. Among them, the intensification of phenomena of high temperatures, heat waves, and UHIs are to be considered when monitoring indoor radon in a city, since the data collected exhibit peculiar features, reflecting those particular conditions.

The aim of the present work is the monitoring of indoor radon concentrations at different floors of a multi-storey apartment building, located in the historic center of Rome (Esquilino) and built in the late 800 s, when tuff was still commonly used in construction. In a previous study (Soldati et al. 2022a), indoor radon had been monitored at the I and the VI floor of a nineteenth-century building in this same area for 1 year. The study found higher radon values at the highest floor during the cold season, with an inversion of the trend from June to October, and markedly different radon levels in the rooms of the same house. These results indicate a major role of anthropogenic factors (such as heating and ventilation), able to produce air convection through the stack effect, and motivated us to carry out a new set of measurements to study in more detail the dynamics of radon transport throughout all floors of a similar building, and how it is influenced by indoor and outdoor air temperature. We analyze the time series over a whole year, to reveal similarities and differences in radon levels between the dwellings and along the seasons. In addition, we discuss the specificity of indoor radon data collected in a very urbanized area of a big city and introduce the issue of the relationship between indoor radon and UHI effect.

## Materials and methods

### Experiment design

**Fig. 1:**
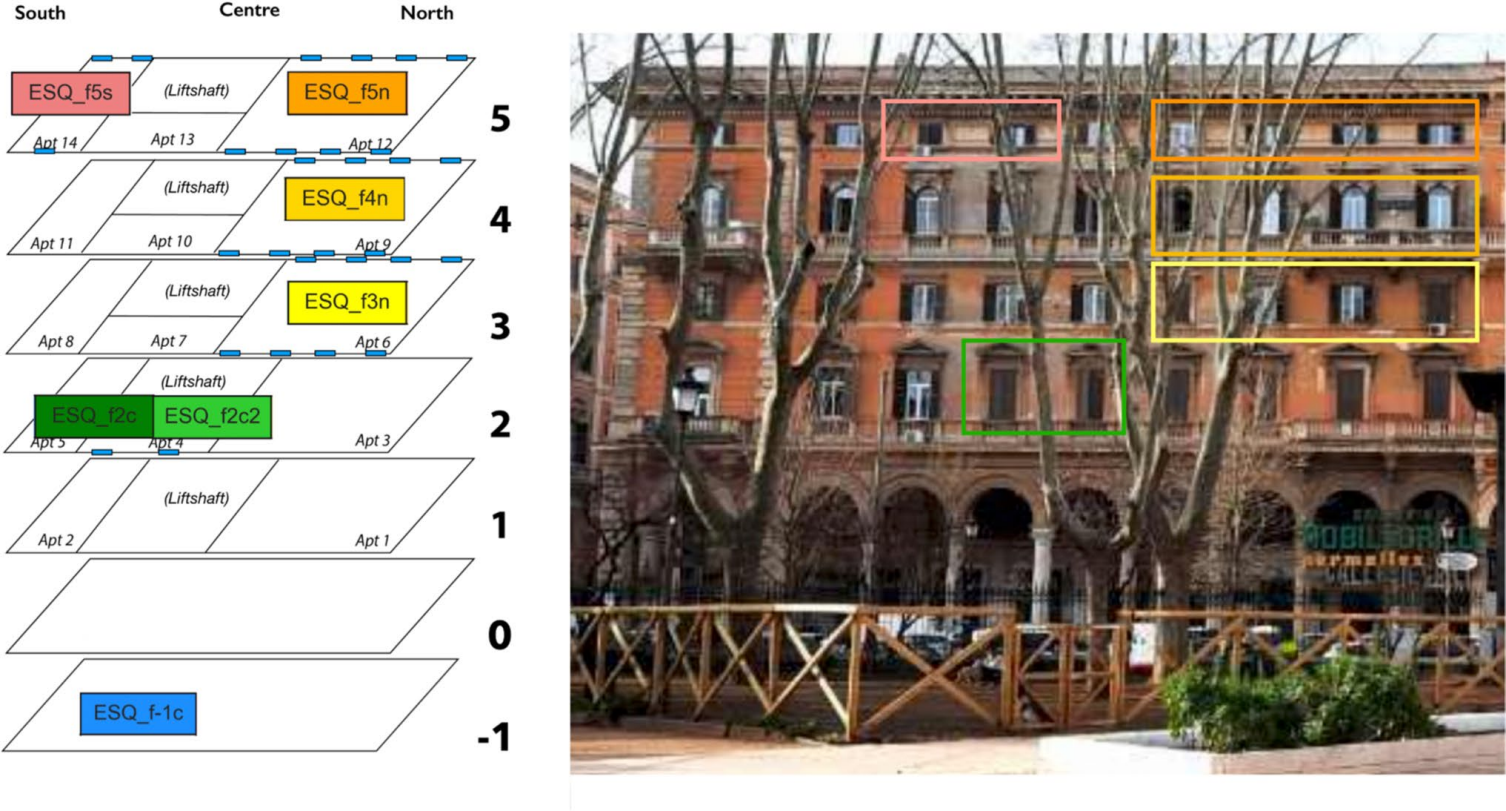
Location (left) of the radon detectors in the monitored building (right) in the Esquilino historical quarter

The experiment was entirely conducted inside a multi-storey residential building in the Esquilino district, where six apartments at five different floors (the basement and the floors 2–5) were monitored for about a year by means of seven commercial solid-state radon detectors. Each floor consists of three apartments, one facing south, one facing north, and one located in the middle. The instruments are named after this location adding “n”/ “s”/ “c” to the floor number (“f2”, “f3”,..); a sketch of their location is provided in Fig. 1. It shows that all the floors have been monitored but the ground level, which hosts some shops, and the I floor, entirely owned by the same tenant. At the V floor, two apartments are investigated (Apt. 12 and Apt. 14 in Fig. 1), while two instruments were installed in different rooms of an apartment (Apt. 4) at the II floor. Figure 1 also shows the location of the lift shaft in the building, which may constitute a preferential access for the upward gas diffusion. The apartments under study differ for exposure, dimensions, window number, and position (light blue in figure); all these factors might have an effect on the measured indoor radon concentration. Another important element to consider is the type of room hosting the instruments; sleeping rooms are expected to provide more stable indoor conditions, while premises like entrance halls or living rooms are supposed to be subjected to a higher influence of occupants. Whenever it was possible, we chose to install the radon detectors in the sleeping room; the sole exceptions are ESQ_f5n and ESQ_f4n.

**Fig. 2:**
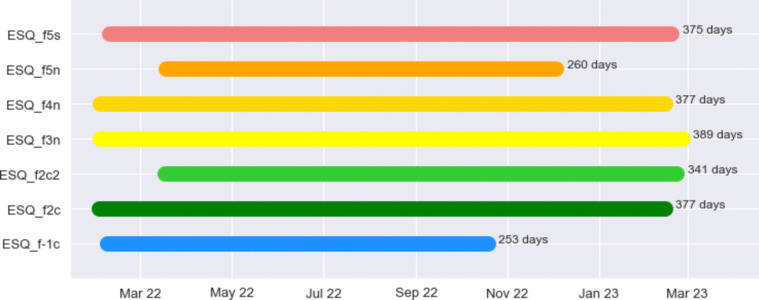
Recording intervals of the instruments employed in the experiment

The availability of long and continuous time series, ideally with no gap, is crucial for monitoring radon variations on both short and long timescales, and to detect periodicity from semidiurnal to seasonal length. The intervals over which the instruments operated are listed in Fig. 2 along with the length (no. of days) of the time series collected.

Five detectors were installed in the first week of February 2022, and the remaining two on March 17. They were supposed to collect data for a whole year, and indeed, all the time series are about 365 days long. The exceptions are the instrument in the basement, which stopped working first, likely due to the high level of humidity which can reduce the duration of the battery, and one of the two located at the V floor, for unknown reasons.

### Instrumentation

**Fig. 3:**
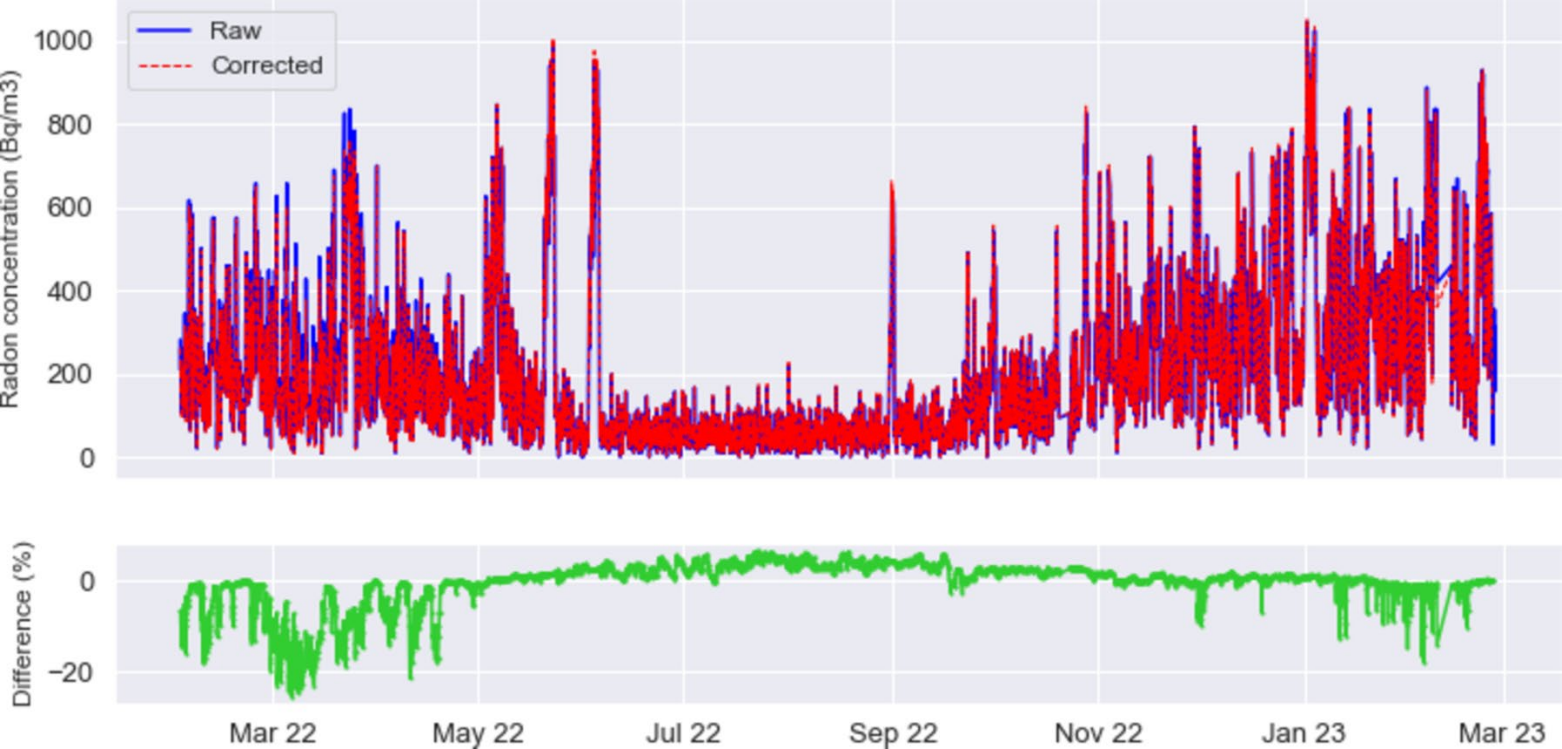
Radon concentration measured at the III floor (top): blue/red/green lines refer to raw radon data, radon data corrected for the specific instrument response, relative humidity and temperature and their percentage difference (bottom)

The instruments adopted are small-sized commercial solid-state radon detectors, produced by the company Algade, model “AER C,” that, besides radon concentration, simultaneously acquire local temperature and relative humidity and whose efficiency has been tested over decades of use within the IRON network (Cannelli et al. 2018). Despite originally being designed for consumer applications, their performances resulted comparable to those granted by devices specifically designed for scientific applications: as a matter of fact, a customized correction function has been derived for each sensor according to a characterization procedure employed at the INGV Radionuclide laboratory to meet the desired standards (Galli et al. 2019). The correction formula R(s,RH,T), as a function of the specific instrument response (s), relative humidity (RH), and temperature (T) AER readouts, ensures data correctness and reliability in all operating conditions; it is worth noting that humidity affects the collection of short-lived alpha emitting radon daughters onto the solid-state detector. To give an idea of how much this correction may affect the data, we provide in Fig. 3 an example of a raw radon time series (blue) and the same time series after correction for R(s,RH,T) (red). Radon data are recorded at the III floor, where although the degree of humidity and temperature are not particularly high, they might account for up to 20% of the radon value. This discrepancy is observed during the coldest months (January to April), while in the warm season, the radon variation ranges between 0 and 5%. The conditions of RH and T appear similar at the other floors of the building and so does the extent of the correction of raw radon data; only in the basement it increases up to about 40% in August and September, when relative humidity and temperature reach their maximum values.

Further details on the sensitivity, measurement error and single instrument characterization are described in previous studies (Galli et al. 2019; Soldati et al. 2022a). It is worth saying that in the framework of time series analysis, the instrument uncertainty on the single measurement is not so significant in characterizing the accuracy of the series itself because it only represents an upper bound of the true error, which is all the more unreliable the more data in the series; for this reason, it is not shown on the plots. The range of temperature and humidity in which the detectors operate correctly is provided by Algade. The acquisition time (sampling rate) may vary in the range 15–240 min; we chose to set it to 120 min, fully sufficient to allow the detection of time variations with semidiurnal period, the shortest significant periodicity observed in this kind of data and, at the same time, long enough to grant acceptable precision down to about 100 Bq/m^3^ or less concentration levels (Barberio et al. 2018; Kumar et al. 2015; Piersanti et al. 2015; Richon et al. 2009; Siino et al. 2019; Zafrir et al. 2013).

It is important to notice that the employment of active devices (that record real-time continuous measurements) for analyses of indoor radon in residential environments is quite a rarity: in general, they are used to provide short-term diagnostic measurements during radon mitigation strategies, and for initial verification of their success. To our knowledge, most of the published analyses on residential radon monitoring made use of passive alpha-track detectors, generally deployed for two consecutive 6-month-long intervals in the cold (fall-winter) and warm (spring–summer) seasons. As a consequence, they usually provide averages of the annual radon concentration and the fraction of dwellings with radon concentration exceeding any reference level. Even the indoor radon surveys of the European countries, including the Italian one (Bochicchio et al. 2005; Leonardi et al. 2021), were performed with passive measurement techniques (Pantelić et al. 2019) except in one country (Cyprus), where high sensitivity active portable radon monitors—RADIM3A (Anastasiou et al. 2003; Theodoulou et al. 2012)—were used. Recently, Tunyagi et al. (2020) introduced a promising prototype for the continuous real-time monitoring of Indoor Air Quality, that can simultaneously measure radon, CO2, CO, VOCs and environmental parameters, and that is currently under testing.

### Data processing

Before starting the analysis, the collected data needed to be preprocessed. The first step was data cleansing: time series of radon concentration, local temperature and relative humidity were imported and processed with the pandas software (a Python library), and the continuity of time series was checked via a search for potential “NaN” values, gaps, and outliers. Different statistical tools were employed: arithmetic mean as an indicator of central tendency, standard deviation to measure the amount of dataset dispersion/variation, rolling mean or moving average to smooth out small fluctuations and gain insight into trends by filtering higher frequency components. Data were further corrected for the effect of the varying degree of humidity to which instruments can be exposed, as detailed in the previous section.

The correlations between pairs of radon time series were quantified using Pearson correlation coefficients, which can be seen as a normalized measure of covariance. Since radon data from different instruments are very rarely synchronous, we shifted the times of data recording of the two stations to a same/shared clock, resampled the data at 1 h, and finally performed a linear interpolation to get more observations at the same clocks for the pair of stations considered. Furthermore, cross-correlations were calculated between outdoor temperature data provided by the weather forecast website 3bmeteo (https://www.3bmeteo.com) on a daily basis and radon and internal temperature data measured setting the acquisition time to 2 h, after aggregating them into 1-day frequency time series.

## Results

### Radon time series

**Fig. 4:**
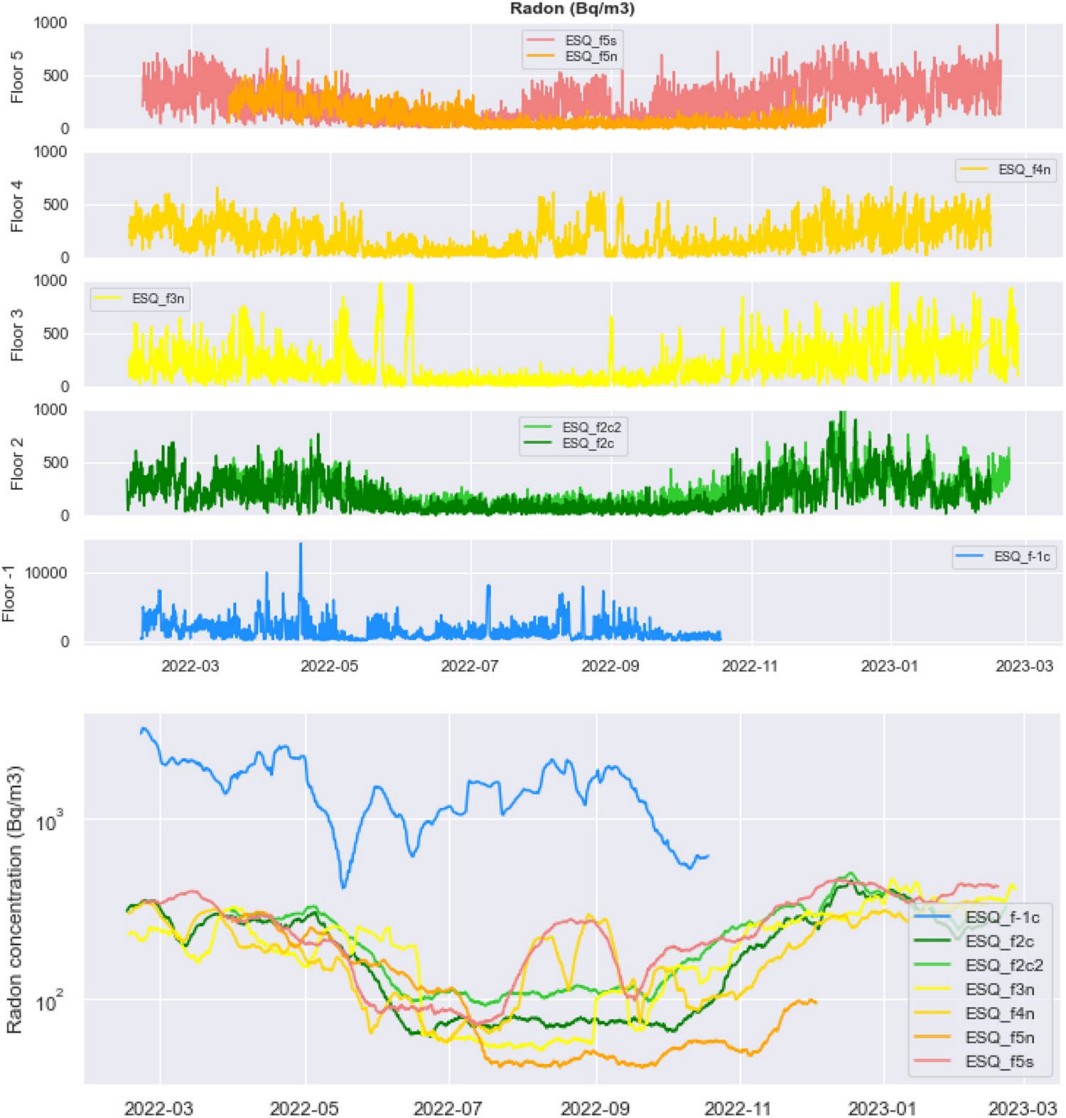
Radon concentration recorded by the seven instruments distributed over five different floors (top). At the II floor and the V floor, two instruments recorded radon data simultaneously. Two-week rolling mean of radon concentration at all the instruments (bottom)

Figure 4 (top) shows the time series of radon concentration recorded in the apartments at the various floors and corrected for humidity and temperature as discussed in “Materials and methods.” The bottom panel includes in the same plot the 2-week rolling mean of all the above data on a logarithmic scale (to be able to compare radon values with very different amplitudes such as those observed in underground environments, e.g., the basement).

Both the panels reveal that, as expected, the highest indoor radon concentration is observed in the cellar at the basement, indicating that the main contribution of radon activity comes from the ground and that this space may suffer from scarce ventilation. Radon level appears almost uniform at the other floors, with no apparent damping in upward gas diffusion, so higher floors do not always correspond to lower radon value, that is to say, as already noted (Borgoni et al. 2014; Soldati et al. 2022a), elevated floors are not safer than lower floors.

On average, radon concentration measured indoors is higher in fall-winter than in spring–summer for all the deployed instruments, as observed in many cases worldwide (Belete and Shiferaw 2022; Hadad et al. 2007; Kellenbenz and Shakya 2021; Papaefthymiou et al. 2003; Soldati et al. 2022a, 2022b). Indoor radon levels are frequently at the highest in the colder months likely because of tightly sealed homes and the thermal stack effect. Due to the larger indoor-to-outdoor temperature difference, cold temperatures increase the pressure gradients towards the building, so that more air is being pulled in from the ground, which elevates the risk of radon entering the home. On the other hand, anomalous peaks are observed at the IV and V floors in August and on the III floor in June, and seasonal fluctuation of indoor radon is not clear in the cellar at the basement, which is probably the less visited area of the building, with consequent little or no air exchange with the outdoors (and constant along the year). These observations may suggest that radon concentration would be more affected by residents’ habits rather than purely meteorological factors. Apparently, when occasionally an apartment remains closed for some consecutive days in summer, radon concentration increases to the level observed in wintertime.

Table 1 shows the descriptive statistics of the radon time series recorded over three time intervals: 1 year (top); the cold months, November to April (middle); and the warm months, May to October (bottom). The parameters computed are the mean, minimum, and maximum values of radon concentration data recorded by each instrument, its standard deviation, and the number of data employed in the calculations (“count”). The main observation is that the average indoor radon concentration in the summer is about half the winter value at all floors of the building. Maximum radon levels instead decrease by less than 30–40% from the cold to the warm season.

**Table 1:**
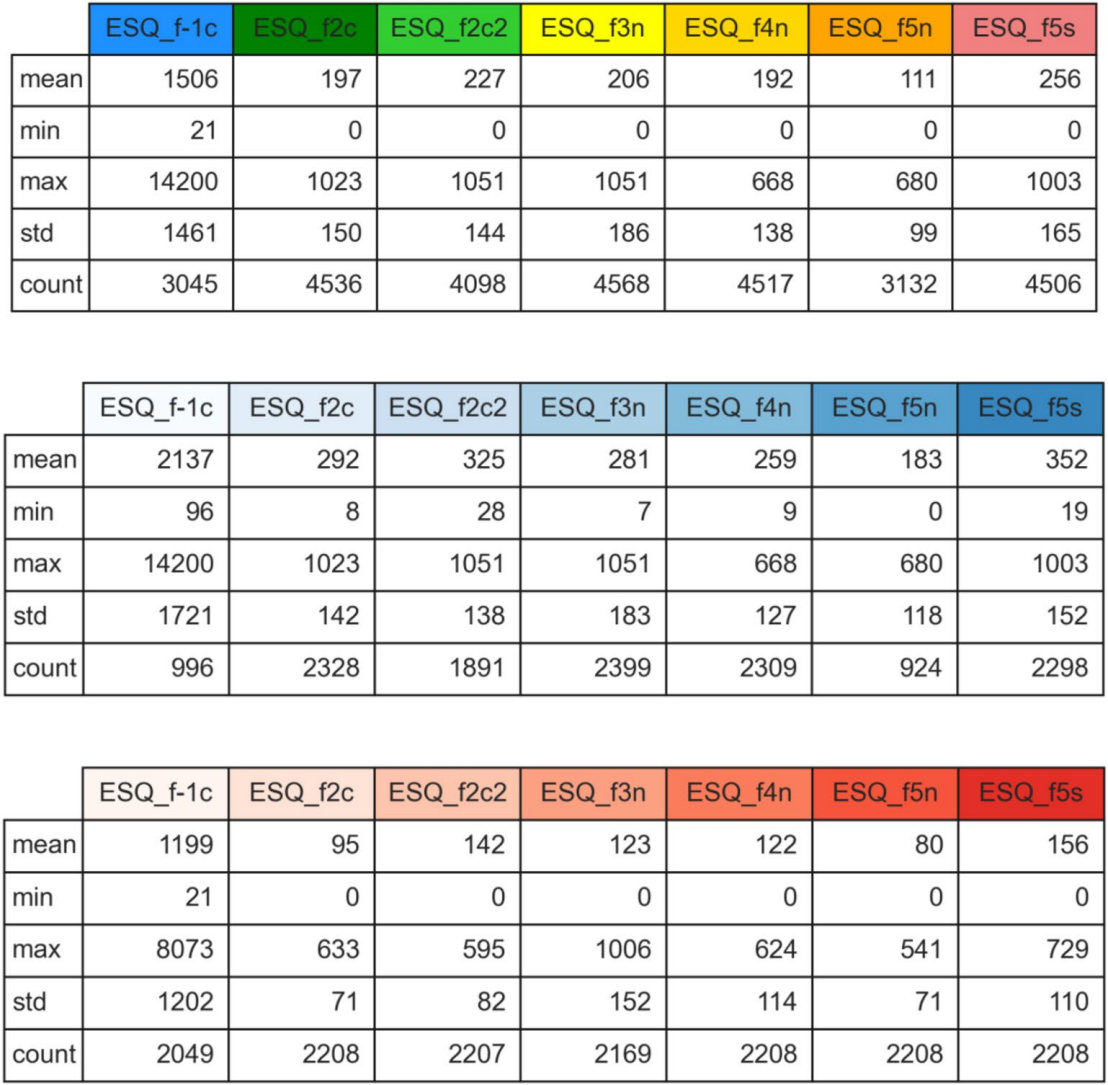
Descriptive statistics of the radon time series recorded over the whole length of the experiment (top), during the cold season (November to April, middle) and during the warm season (May to October, bottom)

### Radon periodicity and temporal changes

**Fig. 5:**
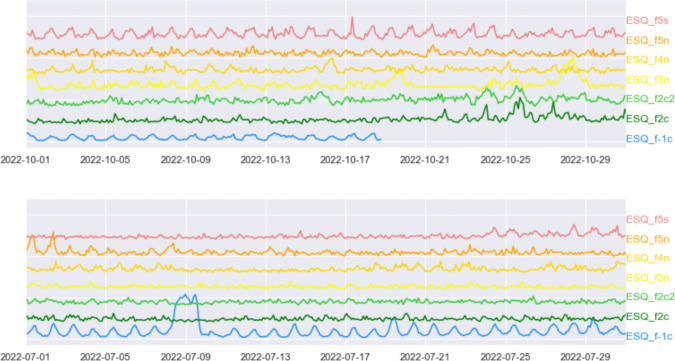
Normalized radon concentration recorded by the seven instruments of the experiment during the month of October 2022 (top) and July 2022 (bottom)

As anticipated in the previous sections, employing active devices for radon measurements allows us to study the temporal variation of the gas in an indoor environment on multiple timescales. We have seen that, on the longest scale, indoor radon concentration shows seasonal fluctuations (with radon higher in the cold season) on all inhabited floors while this behavior is less evident in the basement, which can be considered a less disturbed environment, as it is rarely frequented. Incidentally, we note that this evidence could be, at least partially, linked to a shorter time window of available data for this floor. By choosing an autumn month (October) and a summer month (July) as an example, we can observe that October 2022 radon concentration data in Fig. 5a reveal a clear diurnal periodicity at the lowest (− 1) and highest floor (the fifth), which is not so evident at the intermediate storeys, nor during the month of July as shown in Fig. 5b.

The power spectral density calculated from the entire time series of radon shows a peak at the period of 1 day at all the instruments, but the spectrogram indicates that the high energy at this period is persistent throughout the whole duration of the experiment only in the radon time series recorded in the basement (Supplementary Fig. 1). The signal is low at levels II–IV, and it is not persistent at the V floor (especially at instrument ESQ_f5n), where it tends to decrease in the warm season (and with apparently different behavior in the two apartments monitored at that floor). The 1-day periodicity is well recognized in literature, and it is thought to be a consequence of the diurnal pressure and temperature cycle (Barberio et al. 2018; Kumar et al. 2015; Piersanti et al. 2015; Pinault and Baubron 1996; Schery et al. 1984; Siino et al. 2019).

**Fig. 6:**
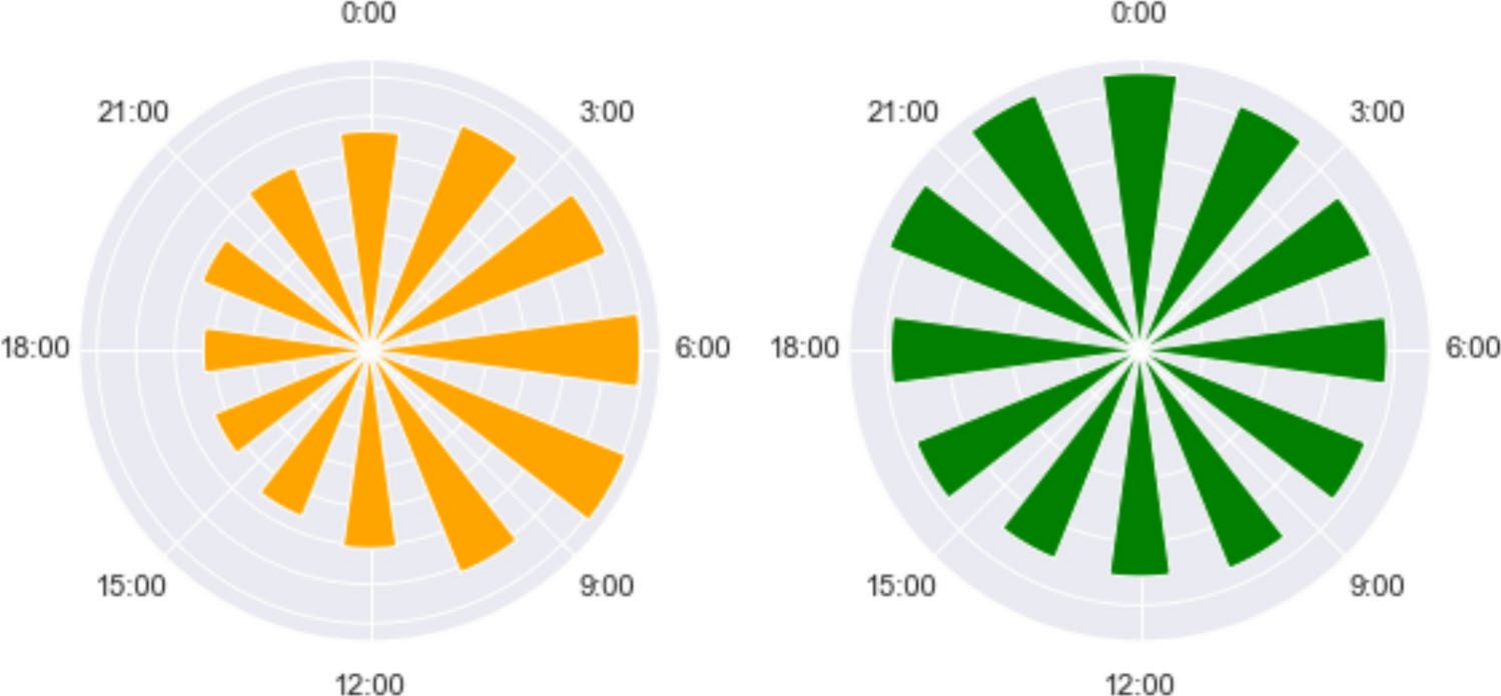
Average indoor radon as a function of hour for instrument ESQ_f5n at the V floor (left) and ESQ_f2c at the II floor (right)

Active radon monitoring may also help to study the temporal variability of indoor radon activity during the day (and the night). Figure 6 shows the gas concentration as a function of the hour at the V and II floor, which, in light of the results obtained for each instrument, can be considered representative of the remaining floors: radon level tends to reach its maximum around 9–10 a.m. at all the building’s floors (including the basement) but floor 2: instruments ESQ_f2c and ESQ_f2c2, located in different rooms of the same apartment recorded maximum radon values at night, between 9 pm and 1 am. A reason for this discrepancy may be the different life habits of the residents: we have in fact observed in a previous experiment in an apartment building (Soldati et al. 2022a) that the differences in the radon concentrations measured in dwellings can be only partially ascribed to the spatial distribution of radon emanation from underground and rather to specific characteristics of the houses and anthropogenic factors like hours of presence at home, use of heating systems, and frequency of ventilation, as demonstrated by the rather different levels of indoor radon observed in different rooms of the same apartment.

**Fig. 7:**
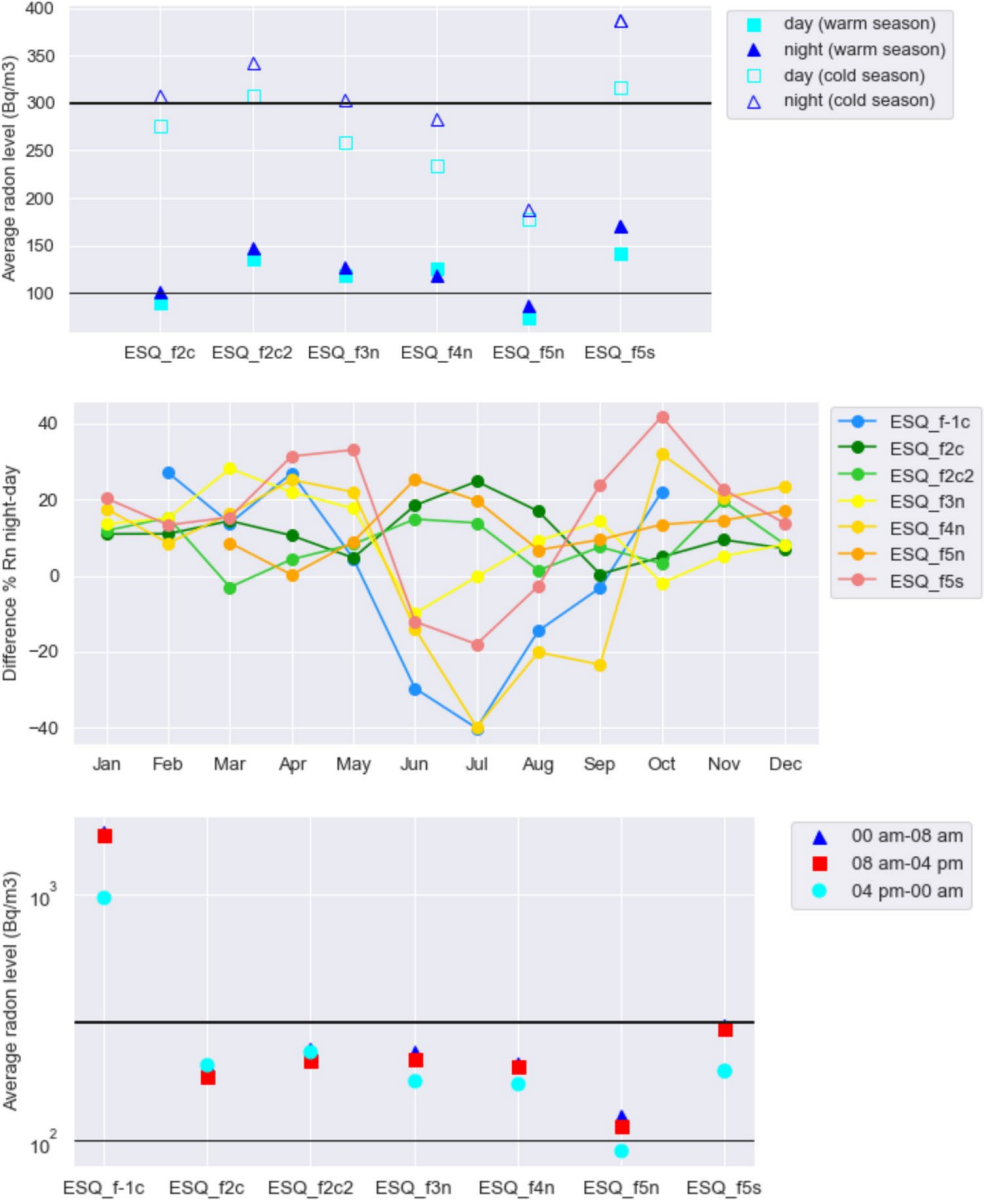
*Top:* Average radon concentration measured during daytime and nighttime by the seven instruments, divided by season. The EURATOM—recommended threshold of 300 Bq/m^3^ and the WHO—recommended threshold of 100 Bq/m^3^ are indicated as black thick and thin lines, respectively. *Middle:* Difference % between average radon concentration measured during the night and during the day for each month of the year. *Bottom:* Average radon concentration measured during three 8-h intervals

Another interesting issue to investigate, given the prevalent diurnal periodicity of indoor radon, is whether there are differences between radon levels recorded during daytime and nighttime, being overnight radon more relevant for residents’ exposure. Splitting each day into day and night and averaging radon concentrations over these intervals, we find (Fig. 7, top) similar radon values, albeit slightly larger at night, during the warm season (May–October), with more pronounced differences in wintertime. This is in agreement with the results of our previous experiments (Gruppo IRON and Classe 3I Liceo Cavour 2020; Soldati et al. 2022a), where we found a predominance of higher nighttime radon in the 30 dwellings located in the urban and suburban area of Rome, albeit with slight difference compared to radon measured during the day. In addition, our results also confirm the finding (Soldati et al. 2022b) that the higher the average radon level (e.g., in fall/winter), the larger the difference between radon values observed at daytime and nighttime, so the active monitoring results even more effective when more pronounced is the health risk for the residents.

The definition of “day” and “night” requires an assumption: here, we arbitrarily chose to define the interval daytime as 9 am–9 pm; testing alternative 12-h intervals like 7 am–7 pm, 8 am–8 pm, and 10 am–10 pm does not change the result. Repeating the test for each month separately reveals that overall, daytime radon and nighttime radon differ substantially only in the coldest months (January–February), with radon invariably higher at night. Examined separately (Fig. 7, center), the single apartments behave differently from each other in terms of radon variations, with maximum differences in daytime-nighttime radon (of the order of ± 40%) observed in different months. This can be partly an effect of the life habits of the apartments’ occupants, which can be quite diverse especially during the warm season (depending for example on the presence at home, the frequency of ventilation, and the use of a conditioning system).

Splitting the day into three intervals of 8 h each (given the sampling time of 2 h this test is less significant than the previous one) as in Fig. 7 (bottom), the difference in radon is appreciable just in the basement (ESQ_f-1c, where average radon is higher during the day) and at the V floor (ESQ_f5s). This result reflects the findings of the previous section concerning the 1-day periodicity, mainly evident on the radon time series recorded by the same two instruments. The maximum discrepancy in indoor radon concentration is observed between the interval from 4 pm to midnight and the rest of the day at all the instruments but the ones at the II floor, where radon looks constant throughout the intervals considered.

Figure 7 includes the threshold recommended by EURATOM and WHO, of 300 Bq/m^3^ and 100 Bq/m^3^, respectively (European Commission 2013; WHO 2009; Legislative Decree 101/2020 integrated and modified by Legislative Decree 203/2022). They are named “action levels” because they represent the maximum levels of indoor radon allowed after which some action should be taken to reduce gas concentration. We find that all the instruments measured an average radon concentration between 100 and 300 Bq/m^3^, independently on the definition of “daytime” and “nighttime”; only in the basement (ESQ_f-1c) the indoor radon level substantially exceeded the Euratom threshold.

### Radon and environmental parameters

**Fig. 8:**
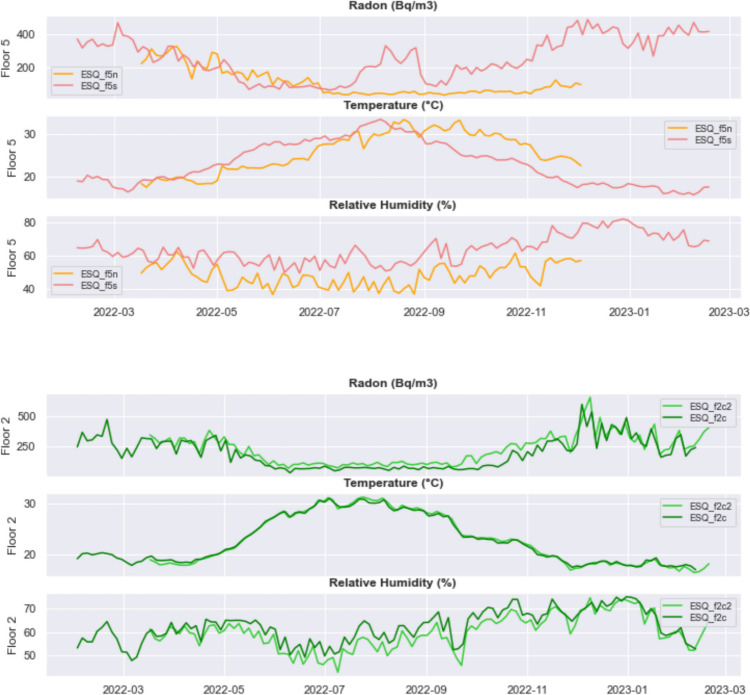
One-week rolling mean of the time series of radon concentration, internal temperature, and relative humidity recorded by the two instruments located at the V floor (top) and II floor (bottom)

Figure 8 illustrates the comparison between the time series of radon/temperature/relative humidity recorded by the instruments located in two different apartments at the V floor and in two different rooms of the same apartment at the II floor.

The two apartments at floor 5 show a difference in the trend of radon concentration, which decreases with increasing temperature up to August 2022 and then rises again at ESQ_f5s, while ESQ_f5n has not yet begun to increase in December, when the instrument stopped working. The curve of temperature vs time in ESQ_f5n (the apartment north facing) looks shifted forward by about 1 month with respect to the one in ESQ_f5s. If the apartment facing North heats up more slowly than the one facing South, we can guess that the radon trend in the two apartments might reflect this delay.

Also, the moisture content may affect indoor radon: humidity, significantly lower in ESQ_f5n, may in fact alter the condition of indoor air because it may lead to a more frequent need for aeration and—as temperature—may induce an increased emission of radon gas from the rocks. Despite the fact that we have corrected the raw data for relative humidity, we cannot rule out its potential effect (not linked to any systematic instrument error), which is not evident from Fig. 8.

At the II floor, the relationship between radon and humidity in two rooms of the same apartment is opposite compared to floor 5: ESQ_f2c has higher humidity than ESQ_f2c2 and lower radon, but in this case, the differences are so small that we should simply consider that the two instruments record the same radon concentration.

**Fig. 9:**
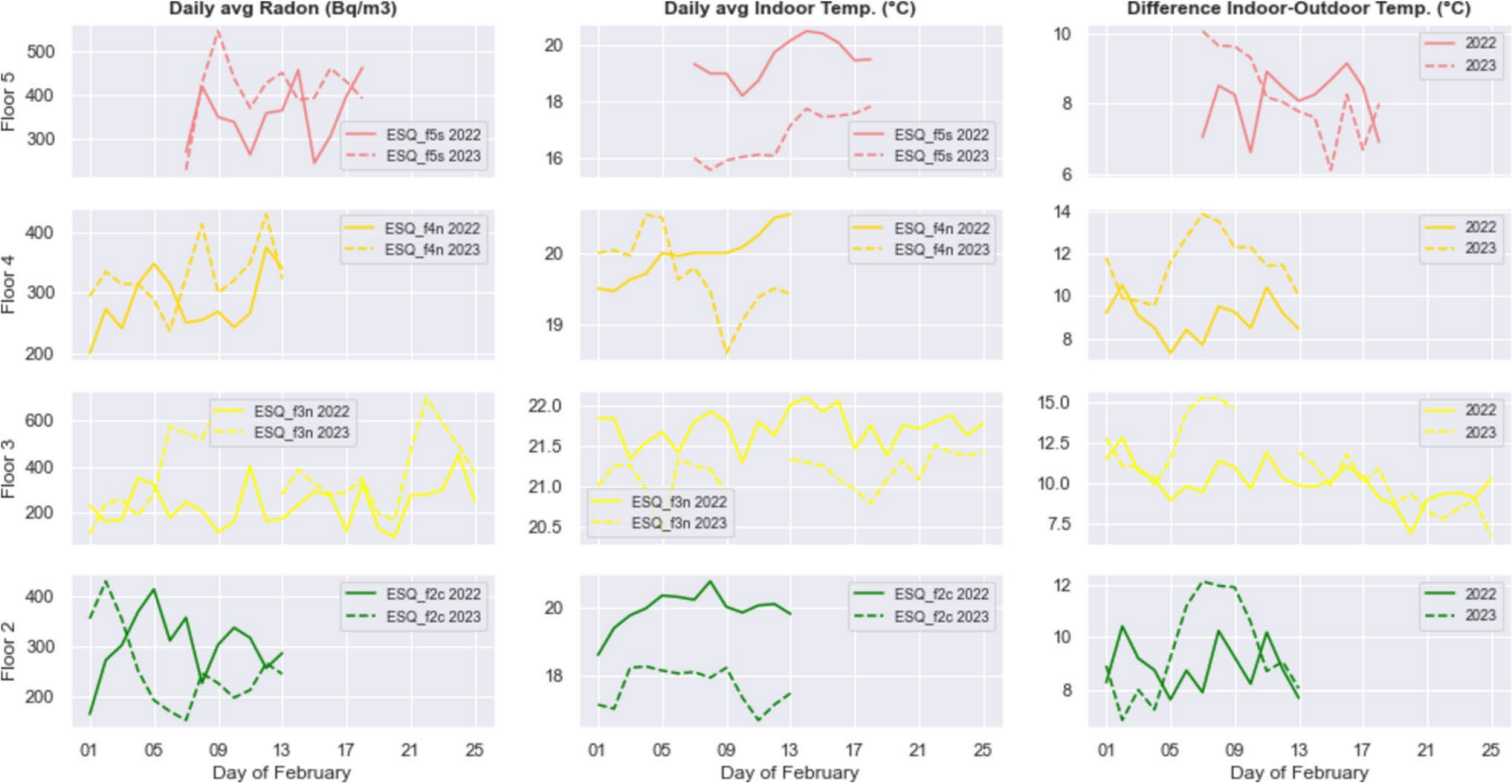
Daily means of radon concentration (left), internal temperature (middle), and indoor-to-outdoor temperature gradient recorded at 4 floors in February 2022 and February 2023

Since the experiment was conducted from February 2022 to March 2023, we have the opportunity to compare the radon data relating to the same month of February for two consecutive years. Figure 9 shows radon, temperature, and indoor-to-outdoor temperature differences recorded at 4 floors in February 2022 and February 2023. While the temperature inside the apartment is recorded by the instruments, the outdoor air temperature has been downloaded by the 3BMeteo weather forecast website https://www.3bmeteo.com. Data are provided as time series with daily frequency by the meteorological station nearest to the study site (named “Roma Esquilino”) and are compared to daily means of radon and internal temperature data we measured.

The comparison reveals that in February 2023, the temperature in the apartments was lower at all floors, while radon concentration was generally higher. This result apparently would disclaim one of the established facts about indoor radon mitigation, namely that limiting the use of heating at home would reduce the indoor radon concentration. In fact, what matters is not the absolute value of temperature inside the dwelling and rather the difference between indoor-outdoor temperature, which is crucial because it is responsible for the gradient in indoor-to-outdoor air density, the major cause of the stack effect (or chimney effect) observed in closed environments. When the outdoor temperature is substantially colder than the inside temperature, the warmer, indoor air is buoyant and convects upward to exit the building creating negative gradient air pressure in the lower levels. The greater the thermal difference, the greater the airflow and the buoyancy force, which favors the entry of outdoor air through cracks in the basement and enhances the exhalation of radon gas from the ground. Despite the small number of data in Fig. 9 and the fluctuating trend of indoor-to-outdoor temperature difference limit the robustness of the conclusions, the hint we can get from this comparison is that February 2023 was a colder month than February 2022, and due to the higher temperature gradient, the radon levels observed in that month overcome the ones of 2022. The exception is floor 2, where a higher indoors-to-outdoors temperature difference corresponds to a lower radon level in most of the few days of recordings (3–13 of February).

**Fig. 10:**
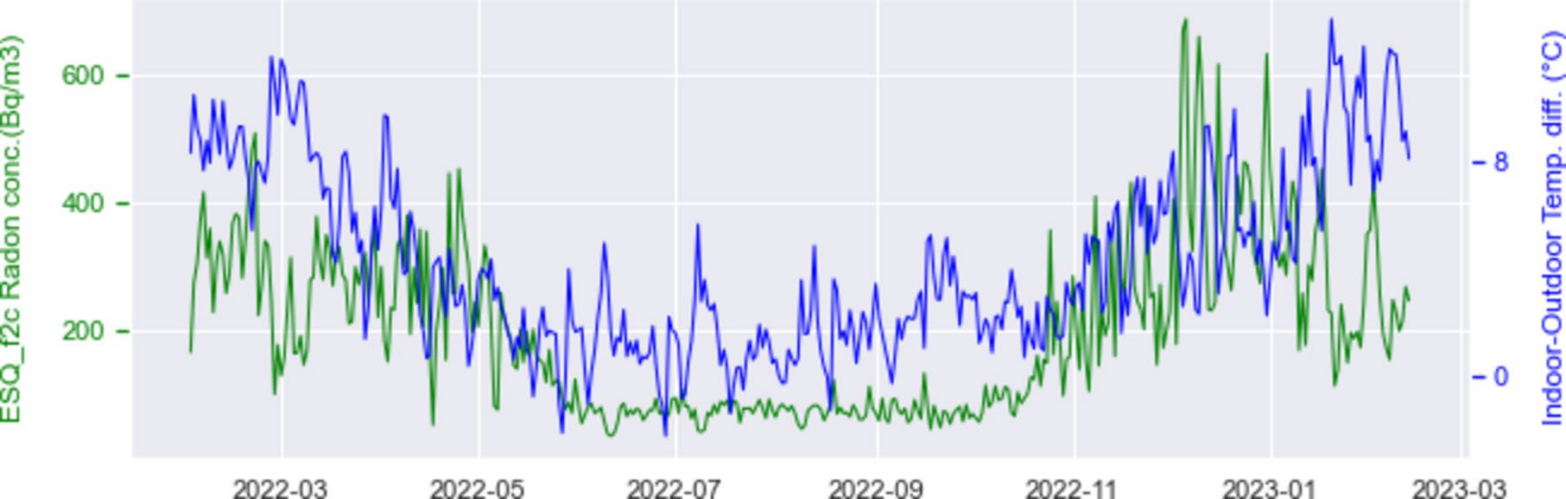
Daily means of radon concentration and indoor-to-outdoor temperature difference recorded at the II floor (ESQ_f2c) over the whole duration of the experiment

Extending the analysis to the whole time duration of the experiment (from February 2022 to March 2023), we show in Fig. 10 the daily averaged radon concentration observed at the II floor (ESQ_f2c) along with the indoor-to-outdoor temperature gradient.

The two curves look positively correlated on the long period, while the comparison on a period of 1–2 weeks reveals a relation of inverse correlation. The long-term correlation between radon and temperature gradient is a result which holds true for all the instruments but ESQ_f5n, where we had already noticed an apparent shift between radon and indoor temperature variations. As for the short scale, the relative behavior of time variations of radon and indoor-to-outdoor temperature difference is far less clear on the data collected by the various instruments, indicating a more complex relation.

### Radon diffusion

**Fig. 11:**
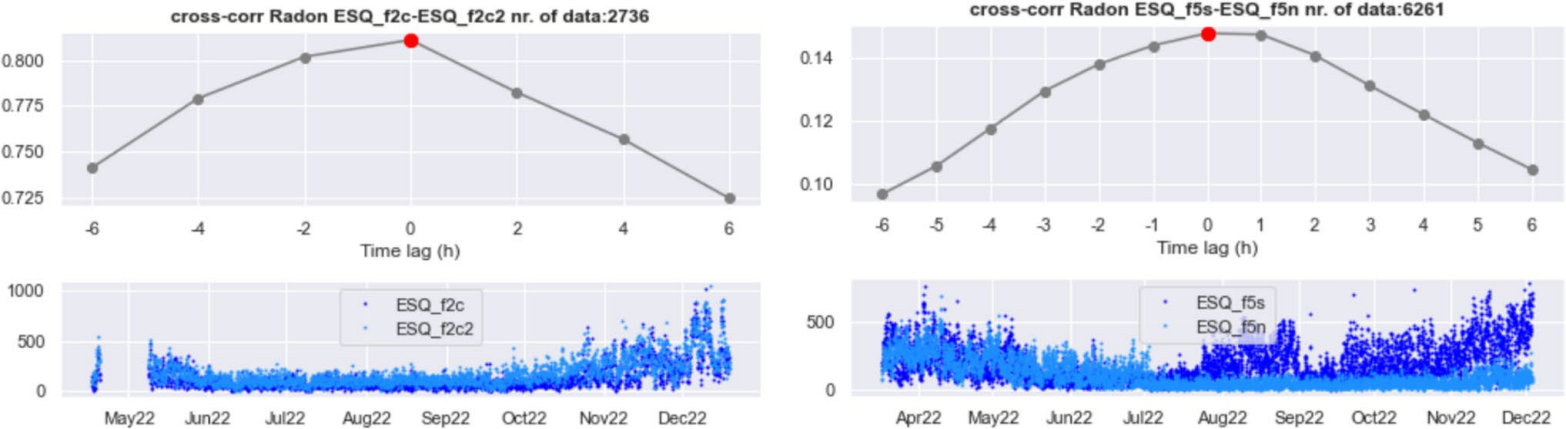
Cross-correlation between radon concentration data of instruments ESQ_f2c and ESQ_f2c2, located in the same apartment at the II floor (left). Radon data employed in the calculation are shown in the bottom panel. (right) Same as (left), with radon data of the two instruments located at the V floor, ESQ_f5s and ESQ_f5n, and cross-correlation computed on data linearly interpolated at hourly intervals

To investigate the dynamics of radon transport, we computed the cross-correlation between the time series of radon concentration of the different instruments. As a first step, we analyze the potential flow of radon gas in the horizontal direction. Figure 11 (left) shows the results obtained for the two instruments located in the same apartment at the II floor (ESQ_f2c–ESQ_f2c2). The radon concentration data used for the computations are sampled every 2 h and shown in the bottom panels, and the cross-correlation is estimated from synchronous data at the two instruments. Not surprisingly, the cross-correlation between radon time series in the same apartment is quite good, and it peaks at 0 time lag.

Repeating the test for the detectors ESQ_f5s and ESQ_f5n, installed in different apartments of floor V, we found no synchronous data; therefore, to get more observations at the same clocks for the pair of instruments considered, we performed a linear interpolation on the overlapping time of the two time series and show the result in Fig. 11 (right). The amplitude of the cross-correlation of radon turns out to be too low to be significant, so we infer that these radon time series do not correlate at all.

Figure 11 seems to indicate that there is no appreciable horizontal movement of radon gas; as for the vertical direction, we repeated the test for all pairs of instruments located at different storeys of the building: no matter how distant in terms of elevation, the value and shape of the cross-correlations are not consistent with a vertical transport of radon. Restricting the calculation of the cross-correlation to the warm or the cold season separately, so that the amplitude of radon concentration values is more uniform, the result is the same.

In a previous experiment, where we monitored indoor radon at various floors of the museum of Rocca di Papa, in the hills surrounding Rome (Soldati et al. 2022b), we could trace the vertical radon diffusion, making a first-order estimate of upward radon velocity. The main difference between the cases explored is the nature of the building studied: a public workplace vs a residential building. The museum is a single entity, with the internal spaces and the storeys communicating with each other so that they are approximately subject to the same conditions of ventilation and heating or conditioning. The apartment building is instead composed of a number of flats, each one under different conditions of aeration, temperature, and humidity, depending on the life habits of the tenants; these factors likely prevent a uniform vertical flow of radon gas.

**Fig. 12:**
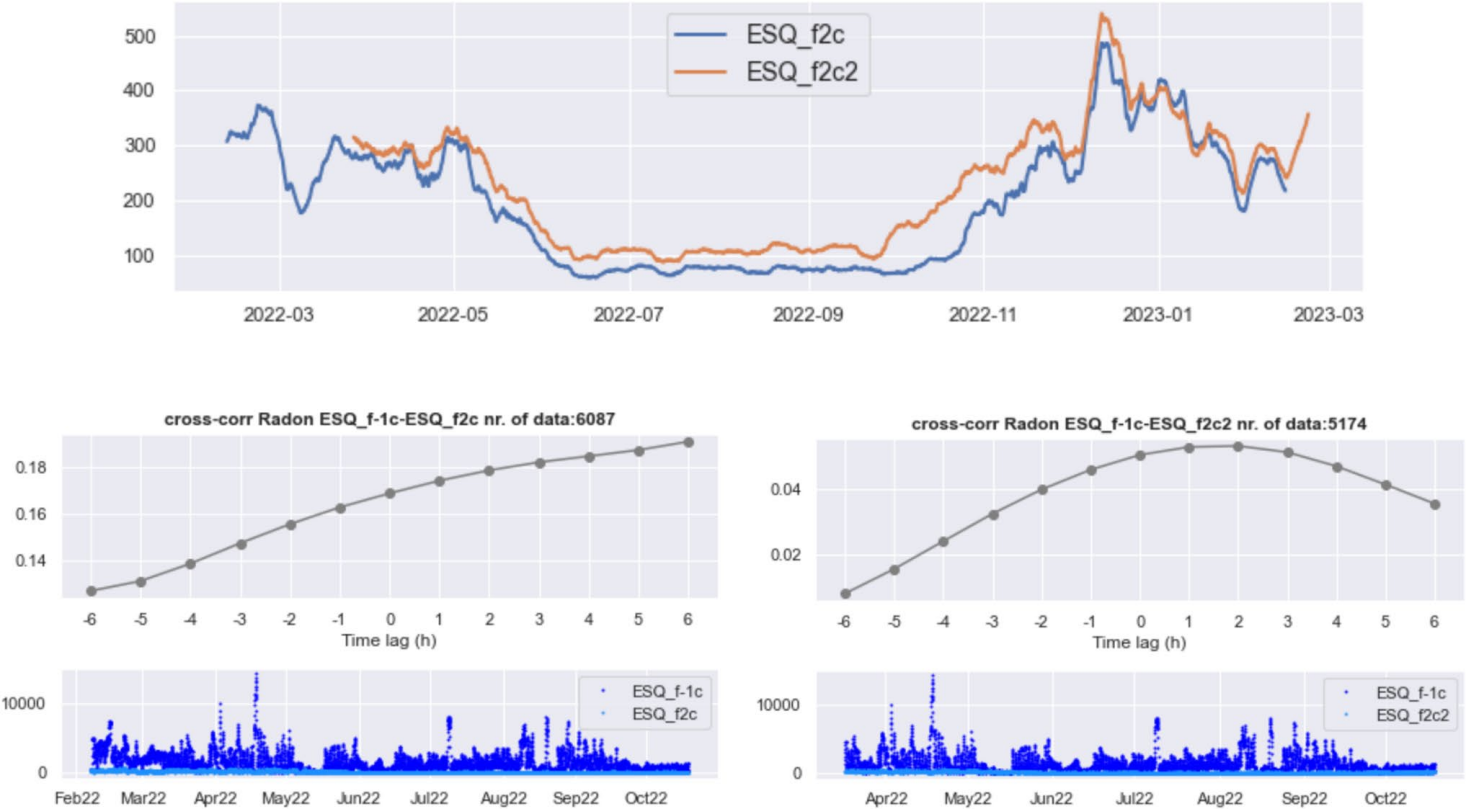
Top: 10-day rolling mean of the time series of radon concentration (Bq/m.^3^) recorded by instruments ESQ_f2c and ESQ_f2c2, located in the same apartment. Bottom: cross-correlation of radon time series measured in the basement (ESQ_f-1c) with ESQ_f2c (left) and ESQ_f2c2 (right)

The 10-day rolling means of instruments ESQ_f2c and ESQ_f2c2 shown in Fig. 12 demonstrates that the similarity between the time series is particularly strong during the cold season where they are almost coincident, while in the warm season, they show a constant offset. Their similarity becomes less clear in October 2022, when ESQ_f2c2 increases while at the same time, ESQ_f2c slightly decreases. This discrepancy starts following an abrupt increase in relative humidity in October 2022 after its strong decrease observed at all the building’s floors (see Fig. 8). It cannot be attributed to the correction for temperature/humidity (Fig. 3). Despite the close similarity between the time series recorded in two rooms of the same apartment, the cross-correlations between each of them with radon in the basement (Fig. 12) differ in shape and the low amplitude makes them meaningless.

Finally, we would note that the results described above about radon horizontal and vertical diffusion may indicate that to better address such questions more resolution is needed (i.e., increasing instrument density and length of recordings or decreasing the sampling time).

### Urban heat islands

As a final consideration about the dataset collected in this experiment, we want to highlight the specificity of the environment where we performed the measurements: the historic district of Esquilino is in fact representative of an urban context, with peculiar exogenous factors such that not only geology, but also temperature conditions may affect the retrieved indoor radon concentrations. The city centers of the metropolitan areas tend to experience stronger heat stress (Copernicus 2020) than their surrounding rural areas, for the vast amounts of heat produced by urban structures, increased land occupation, reduced cooling effect of vegetation, and compounding effect of anthropogenic heat sources. The effect, named UHI, has been known since the seventies (Oke 1973), and its occurrences are rapidly expanding globally in different geographical regions, affecting a growing number of cities (Ren et al. 2023).

In Rome, a recent study (Battista et al. 2023) found that during the whole 2020 year, temperature differences as a result of the UHI were as high as 5.4 °C during the day and exceeded 4.5 °C at night. Considering also the statistically significant increase of temperature of the city between 1989 and 2020 (CMCC 2021), it is reasonable to assume that the consequences deriving from global warming and the effect of the UHI might have a non-negligible impact on indoor radon levels.

**Fig. 13:**
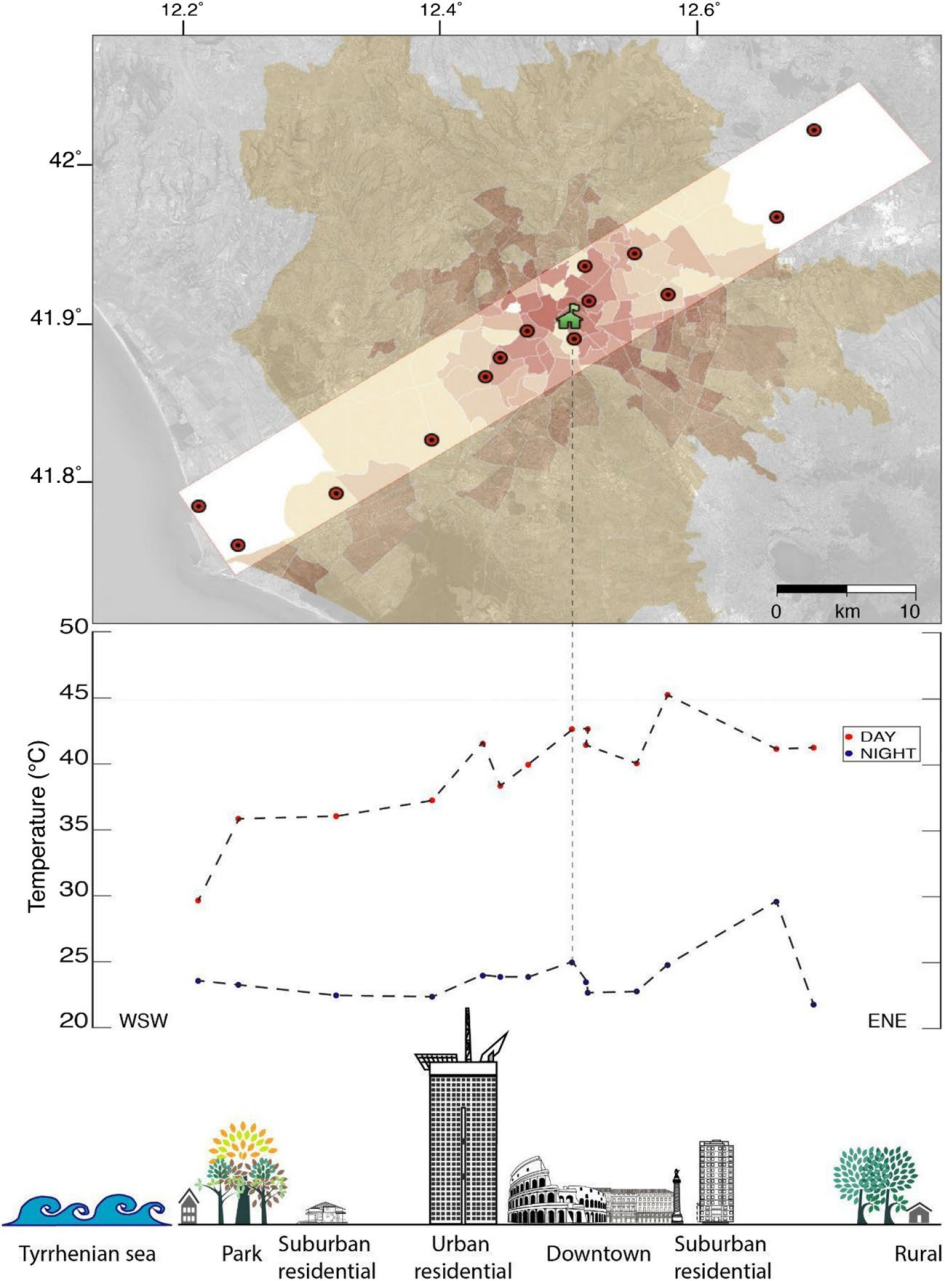
Maximum temperature measured during the day (red circles) and during the night (blue circles) of July 18, 2023, by 14 weather stations located along a WSW-ENE profile crossing the city of Rome and its surroundings (top panel). Darker colors in the map indicate higher building density (ranging from 0 to 44%). The profile extends down to the coast on the West and up to the rural areas to the East. The measure points correspond to the sketch in the bottom panel

In order to delineate a UHI outline, we draw a profile crossing the Roman municipality and the surrounding, non-urban areas for a total length of 50 km, sampling temperatures along it. We selected 14 weather stations from the network https://www.wunderground.com within a box (Fig. 13, top) centered on the location of the Esquilino building (green house) where our radon experiment was conducted and extending along the chosen WSW-ENE profile. We focused on July 2023, as it was the third warmest month of July in Italy since 1800 (CNR-ISAC n.d.), with maximum temperatures observed on the 18th for most of the considered stations (Copernicus 2022).

Red circles in Fig. 13 (center) indicate maximum daytime temperatures, which tend to decrease almost uniformly moving from the city center of Rome towards the seaside (left portion of the curve), where the temperature difference amounts to more than 10 °C. In the opposite direction, the temperature decrease is not so clear (that is why we do not see the expected bell-shaped daily temperature profile). This happens because instead of being somewhat “symmetric,” the selected profile extends from a seaside area (WSW) to a rural one (ENE) with climatic characteristics not so different from those of Rome municipality. As a result, although both the extremes of the profile are classified as rural, they show different climatic characteristics, corresponding to the Mediterranean climate in the coastal strip to the west and to the attenuated continental climate in the internal areas to the east. As for the nocturnal temperature profile (blue circles in Fig. 13), the values are rather similar (ranging between 21.7 and 24.9 °C) along the whole profile. A very slight decrease is appreciable moving away from Rome city center towards the seaside, while towards the rural ENE area, temperatures—rather than decreasing—reach the maximum value (30 °C). Likewise, the daytime temperature shows a maximum (45 °C) at the eastern extremity of the outline instead of in the city center, even if this case could be explained by the high building density (8–15% of the total surface area (Lelo et al. 2016)), indicated by increasingly darker areas in Fig. 13 (top). Many more factors may potentially affect the measured temperatures, like the shape and height of the buildings (called “increased surface roughness,” which can impact airflow), or the amount of greenery, but their investigation is beyond the scope of this analysis. Far from carrying out a quantitative UHI-indoor radon study, which at present is not feasible because of the lack of supporting radon data, our aim is to demonstrate the importance of such a joint analysis because of two reasons: on one side, both the air temperature and the radon concentration in homes may constitute a factor of risk for the respiratory health of the residents; on the other hand, these parameters are closely linked, especially in urban environments, where the UHIs can strongly affect the evolution of indoor air temperature. Given the relevance of the UHI issue for a highly populated city like Rome, we think that this work could lay the groundwork for future investigations, since we are already implementing a high-resolution experiment aimed to make more comprehensive comparisons between indoor radon levels in urban and rural areas.

## Discussion

Differently from the majority of indoor radon analyses, specifically intended to meet the requirements specified in the legislation and conveniently conducted via passive alpha-track detectors (which can only provide average values over the exposure time), we employ here an active monitoring of radon in a residential environment. This allows to study the trend of indoor radon concentrations in great detail, evidencing a rich dynamic in the temporal evolution of detected values in different rooms and floors. The availability of long and continuous time series allows us to monitor radon variations on various timescales and to detect a wide range of periodicity in the data.

In the long term, we find seasonal correlation between indoor radon concentration and temperature recorded inside the dwellings, but a more accurate comparison with the time of occupancy of the apartments indicates that whenever the residents are not home for a period of time long enough (e.g., a couple of weeks), the radon level inside their house gets back to the higher values recorded in wintertime. That would indicate that rather than be caused by a change in radon source, the fluctuations of the gas are more likely to be due to the life habits of the occupants, as confirmed by the temporal trend of radon concentration recorded in the basement, more uniform with respect to the seasonal character of temperature variations. This premise is in fact less “contaminated” by the human presence and thus more representative of the endogenous conditions. On a shorter timescale, indoor radon shows a weak diurnal periodicity, more evident at the lowest and highest floors, maybe due to the greater proximity to the outdoor environment. The active radon detectors employed (and the relatively low sampling rate) also allow to discriminate between daytime and nighttime, an issue particularly critical for the augmented exposure to radon risk during the night, when residents are more likely to be at home. We observe quite similar values of radon concentration during the day and during the night from June to October, while night radon results invariably higher in the fall-winter, in agreement with our previous experiments in the urban and suburban areas of Rome (Soldati et al. 2022a). This result confirms that nighttime radon tends to increase compared to daytime value when or where the radon level is higher; therefore, active monitoring results even more effective in terms of risk mitigation in dwellings characterized by dangerous gas concentration.

Another important remark is that, while we observe the highest radon concentration in the basement (indicating a major gas contribution from the ground), the radon level is practically uniform on all the upper floors, with no apparent damping in upward gas diffusion. Once again (Soldati et al. 2022b, 2022a), we question the commonly accepted idea that the radon hazard be limited to the lowest storeys of buildings, implied in the current legislation regulating the protection against the health risk from exposure to radon.

The design of this experiment, installing instruments at five different floors of the same building, was intended to enlighten the transport dynamics of indoor radon in a residential environment. A similar test conducted in a public building and working place (the geophysical museum of Rocca di Papa, on the Alban hills surrounding Rome) allowed us to make a rough estimate of radon upward velocity (Soldati et al. 2022b). The analysis of horizontal and vertical diffusion of radon inside the monitored residential building does not reveal any uniform or consistent flow of gas in any direction. This suggests that source peculiarities and the life habits on ventilation and, in general, conditioning of the apartments strongly affect the indoor radon dynamics: as a matter of fact, these complexities are able to cancel most of short-term correlations among different apartments in the same building, keeping evident only long-term seasonal ones.

The effect of the environmental parameters like temperature and relative humidity on the time variations of indoor radon is not straightforward, with the exception of the negative radon-temperature correlation on the annual scale. Comparing radon concentration measured in the same apartments in February 2022 and February 2023, the generally lower indoor temperatures recorded in 2023 are accompanied by higher indoor radon levels. This may appear in contrast with the idea that limiting the use of heating guarantees low radon levels; in fact, this happens because despite the low internal temperature, the inside-to-outside temperature differential increases from 2022 to 2023 (and being proportional to the pressure gradient, it enhances the chimney effect and the resultant rate of radon gas exhalation from the ground).

Outdoor air temperature can vary not only temporally but also spatially, with the city center much more affected than the suburban area by the heat waves and the high temperatures characterizing summertime at our latitudes (UHI). This translates into an increase of radon hazard in the historical quarters of Rome, like Esquilino, where the rise in temperature adds up to the already significant risk related to the geology of this area (geogenic radon). Nowadays, over half of the world population lives in urban settlements, and by 2050, two out of three people will live in big cities. Along with the projected trend of global warming, this means that the effect of the UHI on citizens’ health is expected to grow quite a lot in the near future. A joint analysis aimed to understand how indoor radon in urbanized areas is correlated with the UHI would be therefore desirable in view of a reduction of respiratory hazard for the citizens.

## Supplementary Information

Below is the link to the electronic supplementary material.

Supplementary file1 (DOCX 172 KB)

## Data Availability

The dataset analyzed in the current study is available from the corresponding author on reasonable request.
