# Supplementary material for: Assessment of indoor radon levels at multiple floors of an apartment building in the historic center of Rome (Italy): a comprehensive study
Source: Environ Sci Pollut Res Int. 2024 Oct 21;31(52):61660–76. doi: 10.1007/s11356-024-35266-7 (PMC11541404; doi:10.1007/s11356-024-35266-7)
Supplement: Supplementary file 1 — Supplementary file1 (DOCX 172 KB) [file 11356_2024_35266_MOESM1_ESM.docx]

# **Supplementary Material**

# **
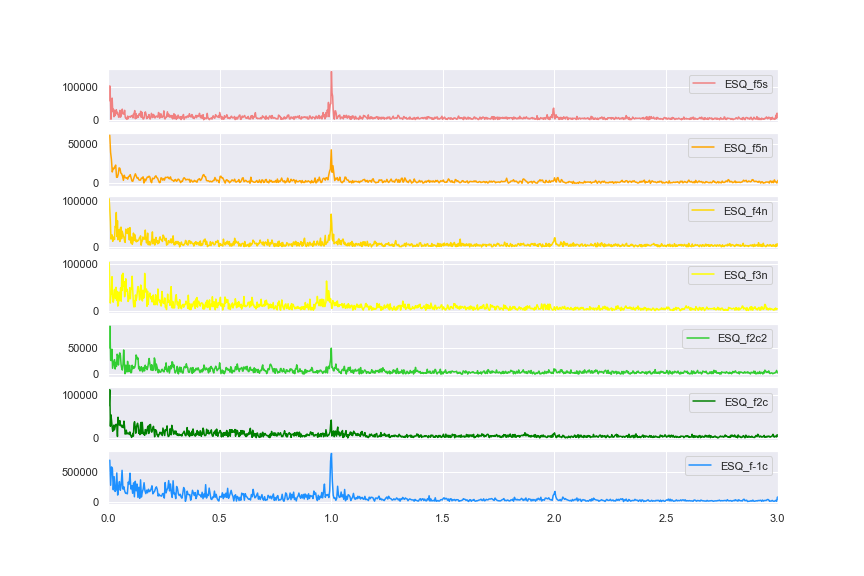

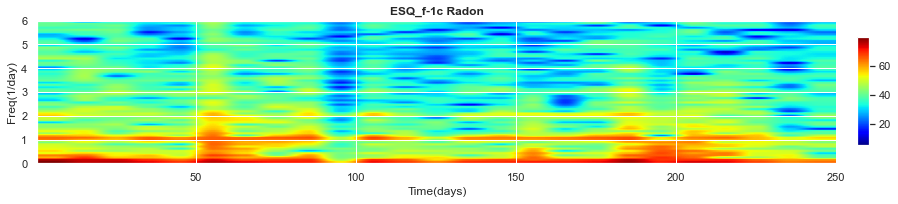
**

**Fig. 1 Fourier (amplitude) spectra of all the time series of radon concentration (top), spectrogram of radon concentration recorded by the instrument located in the basement of the building (bottom). Time is measured in days starting from the first day of recording (7 February 2022).**

# 
